# A computational model to describe multi-regional brain architecture during neurodegeneration in Alzheimer’s disease

**DOI:** 10.1038/s41598-026-54109-8

**Published:** 2026-06-14

**Authors:** Xingfeng Li, Andrea G. Rockall, Paul Edison, Alzheimer’s Disease Neuroimaging Initiative (ADNI), Michael W. Weiner, Paul Aisen, Ronald Petersen, Clifford R. Jack, William Jagust, Susan Landau, Monica Rivera-Mindt, Ozioma Okonkwo, Leslie M. Shaw, Edward B. Lee, Arthur W. Toga, Laurel Beckett, Danielle Harvey, Robert C. Green, Andrew J. Saykin, Kwangsik Nho, Richard J. Perrin, Duygu Tosun, Rachel Nosheny, Melanie J. Miller, Catherine Conti, Winnie Kwang, Chengshi Jin, Adam Diaz, Miriam Ashford, Derek Flenniken, Adrienne Kormos, Michael Rafii, Rema Raman, Gustavo Jimenez, Michael Donohue, Jennifer Salazar, Andrea Fidell, Virginia Boatwright, Justin Robison, Caileigh Zimmerman, Yuliana Cabrera, Karen Crawford, Scott Neu, Naomi Saito, Hannatu Amaza, Matt Glittenberg, Isabella Hoang, Kaori Kubo Germano, Joe Strong, Joel Felmlee, Nick C. Fox, Paul Thompson, Charles DeCarli, Arvin Forghanian-Arani, Bret Borowski, Calvin Reyes, Chad Ward, Robert A. Koeppe, Gil Rabinovici, Victor Villemagne, Brian LoPresti, John C. Morris, Erin Franklin, Haley Bernhardt, Nigel J. Cairns, Lisa Taylor-Reinwald, Virginia M. Y. Lee, Magdalena Korecka, Magdalena Brylska, Yang Wan, J. Q. Trojanowski, Tatiana M. Foroud, Shannon L. Risacher, Hannah Craft, Liana G. Apostolova, Kaci Lacy, Rima Kaddurah-Daouk, Li Shen, David Soleimani-Meigooni, Renaud La Joie, Konstantinos Chiotis, Charles Windon, Julien Lagarde, Jason Karlawish, Claire Erickson, Joshua Grill, Emily Largent, Kristin Harkins, Leon Thal, Zaven Khachaturian, Richard Frank, Peter J. Snyder, Neil Buckholtz, John K. Hsiao, Laurie Ryan, Susan Molchan, Maria Carrillo, William Potter, Lisa Barnes, Marie Bernard, Hector González, Carole Ho, Jonathan Jackson, Eliezer Masliah, Donna Masterman, Nina Silverberg, Lisa Silbert, Jeffrey Kaye, Sylvia White, Aimee Pierce, Amy Thomas, Tera Clay, Daniel Schwartz, Gillian Devereux, Janet Taylor, Jennifer Ryan, Mike Nguyen, Yanan Shang, Lon S. Schneider, Cynthia Munoz, Carlota Conant, Katherin Martin, Kristin O’Leary, Sonia Pawluczyk, Elizabeth Trejo, Karen Dagerman, Mauricio Becerra, Julia Boudreau, James Brewer, Antonio Gama, Jennifer Frascino, Judith Heidebrink, Ronald Petersen, Bradley Boeve, David Jones, David Knopman, Hugo Botha, Jonathan Graff-Radford, Val Lowe, Sudha Seshadri, William Hu, Jacobo Mintzer, Australian Imaging Biomarkers and Lifestyle (AIBL) Study, Christopher Fowler, Stephanie R. Rainey-Smith, Sabine Bird, Julia Bomke, Pierrick Bourgeat, Belinda M. Brown, Samantha C. Burnham, Ashley I. Bush, Carolyn Chadunow, Steven Collins, James Doecke, Vincent Doré, Kathryn A. Ellis, Lis Evered, Amir Fazlollahi, Jurgen Fripp, Samantha L. Gardener, Simon Gibson, Robert Grenfell, Elise Harrison, Richard Head, Liang Jin, Adrian Kamer, Fiona Lamb, Nicola T. Lautenschlager, Simon M. Laws, Qiao-Xin Li, Lucy Lim, Yen Ying Lim, Andrea Louey, S. Lance Macaulay, Lucy Mackintosh, Ralph N. Martins, Paul Maruff, Colin L. Masters, Simon McBride, Lidija Milicic, Madeline Peretti, Kelly Pertile, Tenielle Porter, Morgan Radler, Alan Rembach, Joanne Robertson, Mark Rodrigues, Christopher C. Rowe, Rebecca Rumble, Olivier Salvado, Greg Savage, Brendan Silbert, Magdalene Soh, Hamid R. Sohrabi, Kevin Taddei, Tania Taddei, Christine Thai, Brett Trounson, Regan Tyrrell, Michael Vacher, Shiji Varghese, Victor L. Villemagne, Michael Weinborn, Michael Woodward, Ying Xia, David Ames, Eric O. Aboagye

**Affiliations:** 1 Department of Surgery and Cancer, Imperial College Hammersmith Campus, Du Cane Road, London, W12 0NN UK; 2 The Imaging Department, Imperial College Healthcare NHS Trust, UK Hammersmith Hospital, Du Cane Road, London, W12 0HS UK; 3 Imperial College Memory Research Centre, Department of Brain Science, Imperial College Healthcare NHS Trust, UK Hammersmith Hospital, Du Cane Road, London, W12 0HS UK; 4 University of California, San Francisco (UCSF), San Francisco, USA; 5 University of Southern California (USC), Los Angeles, USA; 6 Mayo Clinic, Rochester, USA; 7 University of California, Berkeley (UCB), Berkeley, USA; 8 Fordham University/Mount Sinai Medical Center, Bronx, USA; 9 University of Wisconsin-Madison, Madison, USA; 10 University of Pennsylvania, Philadelphia, USA; 11 University of California, Davis (UC Davis), Davis, USA; 12 Harvard University/Massachusetts General Hospital, Boston, USA; 13 Indiana University School of Medicine, Indianapolis, USA; 14 Washington University in St. Louis, St. Louis, USA; 15 Northern California Institute for Research and Education (NCIRE), San Francisco, USA; 16 University College London, London, UK; 17 University of California, Los Angeles (UCLA), Los Angeles, USA; 18 University of Michigan, Ann Arbor, USA; 19 University of Pittsburgh, Pittsburgh, USA; 20 Duke University, Durham, USA; 21 University of Texas Southwestern Medical Center, Dallas, USA; 22 Emory University, Atlanta, USA; 23 McGill University, Montreal, Canada; 24 Sunnybrook Health Sciences Centre, Toronto, Canada; 25 St. Joseph’s Health Centre Ontario, Toronto, Canada; 26 Cleveland Clinic, Cleveland, USA; 27 Northwestern University, Evanston, USA; 28 Brigham and Women’s Hospital, Boston, USA; 29 University of California, Irvine, Irvine, USA; 30 Khachaturian, Radebaugh & Associates, Potomac, USA; 31 General Electric, Boston, USA; 32 University of Connecticut, Mansfield, USA; 33 National Institute on Aging/NIH, Bethesda, USA; 34 National Institutes of Health, Bethesda, USA; 35 Alzheimer’s Association, Chicago, USA; 36 National Institute of Mental Health, Bethesda, USA; 37 Rush University, Chicago, USA; 38 University of California, San Diego, San Diego, USA; 39 Denali Therapeutics, South San Francisco, USA; 40 Massachusetts General Hospital, Boston, USA; 41 Biogen, Cambridge, USA; 42 Oregon Health and Science University, Portland, USA; 43 UT Health San Antonio, San Antonio, USA; 44 Rutgers University, New Brunswick, USA; 45 Medical University of South Carolina, Charleston, USA; 46 CSIRO Health and Biosecurity, Clayton South, Australia; 47 Edith Cowan University, Joondalup, Australia; 48 University of Melbourne, Melbourne, Australia; 49 Austin Health, Heidelberg, Australia; 50 Cogstate Ltd, Melbourne, Australia

**Keywords:** Alzheimer’s disease, MRI radiomics, Machine learning classification, Feature selection, Longitudinal study, Mild cognitive impairment (MCI), Neuroscience, Computational neuroscience, Dementia, Alzheimer's disease

## Abstract

We previously proposed an MRI-based machine learning model to describe the mesoscopic architecture of the human brain to aid in classifying subjects as having non-AD related pathology (nADrp) or AD related pathology (ADrp), including mild cognitive impairment (MCI) and Alzheimer’s disease (AD). The method, developed on data from patients scanned at 1.5T showed high performance, but did not generalise well to scans obtained from 3T MRI. In the current work we overcome the problem and extend the approach to patients scanned longitudinally, and at different field strengths. Retrospective T1-MRI data from 1592 subjects scanned at 3T were included to develop the machine learning models. Three additional longitudinal datasets (n = 211) at different magnetic field strengths—1.5 and 3T—were adopted to evaluate the models. Radiomic features were extracted from each brain region. A logistic regression method with least absolute shrinkage and selection operator (LASSO) model selection was employed to classify nADrp from ADrp (classifier 1) or MCI from AD (classifier 2). Classifier 1 that discriminates nADrp from ADrp achieves high performance, with area under the curve (AUC) of the receiver operating characteristics (ROC) of 0.84 in the independent hold-out cross-sectional dataset. High performance was also seen in external testing datasets for classifier 1 (AUC of 0.70 to 0.96). Classifier 2 that discriminates MCI from AD achieves AUC of 0.79 in the independent hold-out dataset and moderate to good performance in the external testing datasets (AUC of 0.56 to 0.93). The new data analysis methods, trained on 3T data, demonstrate potential for aiding AD early detection and disease progression on both 3T and 1.5T scanners.

**Supplementary Information:**

The online version contains supplementary material available at https://doi.org/10.1038/s41598-026-54109-8.

## Introduction

Alzheimer’s disease (AD) is a neurodegenerative disorder characterized by toxic changes in the brain, including abnormal build-up of proteins that form amyloid plaques and tau tangles ^1^. It is the most common form of dementia, and it is estimated that 6.5 million Americans aged 65 and older are living with Alzheimer’s dementia today; it was the seventh-leading cause of death recorded in 2020 and 2021^2^. Globally, it has been estimated that over 46 million people live with dementia, which without effective therapies will increase to 131.5 million ^3^ or 152 million ^4^ by 2050. Currently, there is no cure for AD ^5^, although disease modifying drugs have been approved for patients with mild cognitive impairment (MCI) having a positive amyloid diagnosis ^6^. Slowing progression to AD through early diagnosis and lifestyle changes are important steps for patients ^7^. However, accurate diagnosis of AD depends on the experience of clinical experts, takes several months to years and is costly to healthcare providers. To diagnose objectively and to reduce the burden of the clinical experts, many automated machine learning methods have been developed to classify AD from healthy controls (HC) ^8,9^. While multimodal datasets are increasingly available in AD research, restricting the analysis to T1-weighted imaging constitutes a deliberate methodological choice designed to maximise generalisability and translational relevance. Based on structural MRI from brain, deep learning methods, ^10^ such as fully convolutional neural network ^11^ and radiomics based machine learning methods ^12,13^ were developed to classify AD subjects. There is a wide range of features from MRI radiomics for AD classification ^14,15^, including the most commonly used features such as textures ^16–18^, cortical thickness ^19^ and grey/white mater intensity ^20,21^; of note, the classification performances of these methods have been found to be similar ^22^.

A major challenge of MRI-based machine learning methods for AD classification relates to generalising outputs across field strength. We recently proposed an MRI-based machine learning model to describe the mesoscopic architecture of the human brain and aid in classifying subjects as having non-AD related pathology (nADrp) or AD related pathology (ADrp), including mild cognitive impairment (MCI) and AD ^13^. Based on radiomics from 1.5T T1 MRI, the study showed superiority over routine clinical MRI-based hippocampal volume and CSF biomarkers in diagnosing nADrp versus ADrp. The method showed high performance (up to 98% accuracy) ^13^, but did not generalise well to scans obtained from 3T MRI. This precludes assessment of multiple longitudinal datasets to verify early disease development and progression and dynamic/complexity of this (reverters) and verification of disease in genetically profiled cohorts. There is paucity of machine learning across field strengths, with some studies suggesting variable impact of field strength on sensitivity of radiomics features ^23,24^**.** Confounds were seen with brain parcellation—defining distinct regions within the brain—at different field strengths (1.5 and 3T)^23^ and a need for adjustments when adapting results from one scanner type/field strength or developing multicenter protocols^24^.

On the other hand, other studies report lower field-strength dependent variance when specific regions, e.g. hippocampus, are investigated ^25^ which may be reproducible on test–retest ^26^. Regarding our specific methodology, in using FreeSurfer (a software for 3D surface reconstruction and brain region segmentation)^27^, we anticipate some segmentation differences between 1.5T and 3.0T of 10% and above^28^, consequently impacting radiomics feature values. We rationalized that training algorithms on 3T, with external testing on both 3T and 1.5T datasets will attempt to overcome field parcellation challenges due to the finer resolution at 3T. Thus, appreciating an effect of parcellation on extending utility of multi-regional compartmental radiomics of the brain, we proposed training a new model on 3T data and then extending this model to 3T and 1.5T data. We also increased the sample size for prediction and testing, which was also a limitation of our previous study ^13^.

The purpose of this study was to develop machine learning models using a large set of radiomics features from 3T MRI, and evaluate these models for discriminating non-AD-related pathology (nADrp) from AD-related pathology (ADrp), and MCI from AD; including longitudinal cohorts. As with our previous study, for nADrp subject group, we included healthy brain and diseases unrelated to AD pathology, including Parkinson’s disease (PD) and frontotemporal dementia to better reveal the brain structure changes unique to AD^13^. We hypothesised that machine learning classification models derived from 3T are applicable to 1.5T MRI for AD classification. Additionally, we evaluated these models based on 3 external longitudinal datasets for AD disease progression studies ^29–33^, and predicted that the classification accuracy of these models will vary with disease status fluctuation. We expected to develop a model that has high performance independent of field strength and enables studies of longitudinal and genetically profiled cohorts.

## Materials and methods

### Study population and demographics

**Fig. 1:**
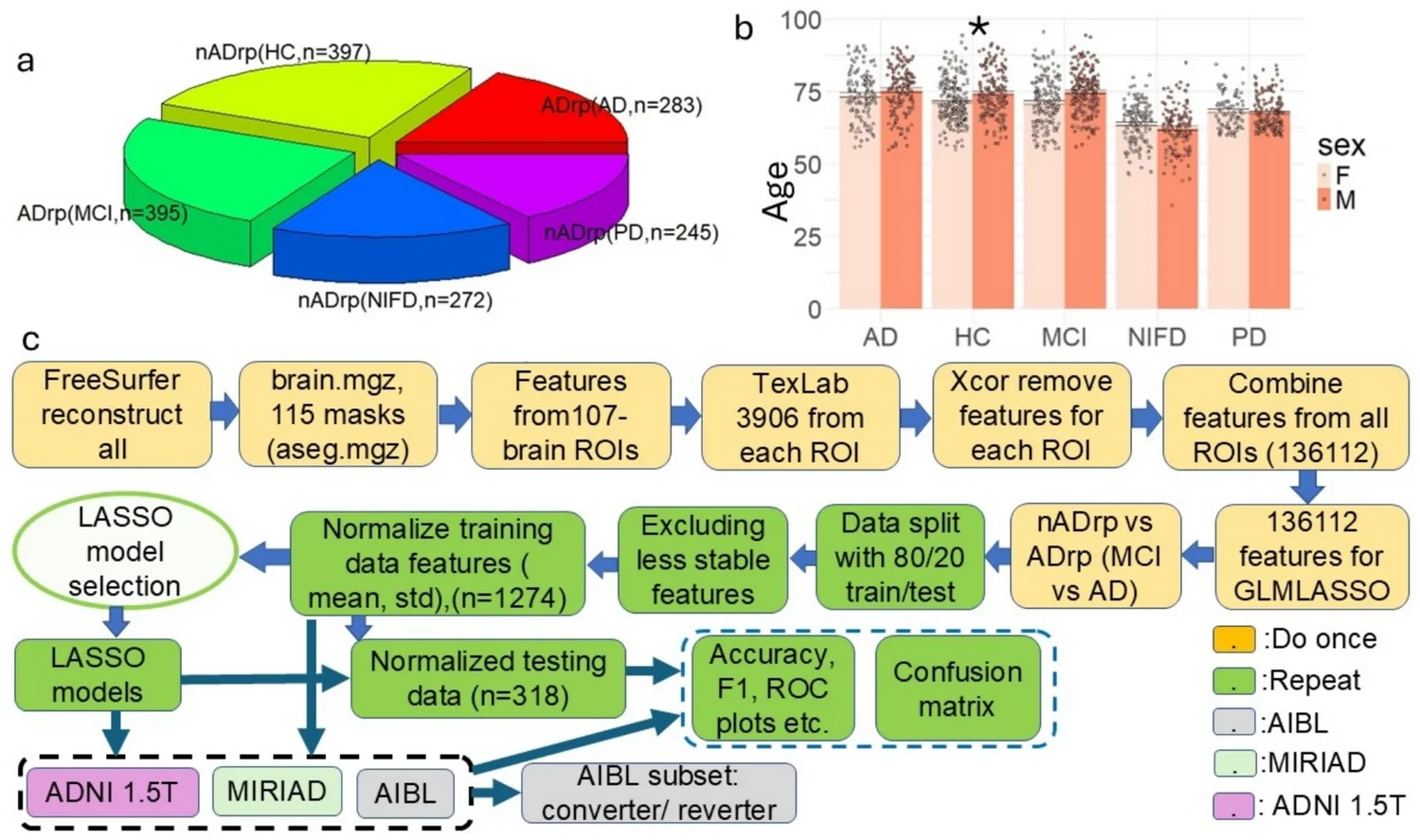
Datasets included in the analysis. (**a**) Pie chart for training and internal testing datasets (ADNI dataset). (**b**) Bar plots for age distribution of the ADNI dataset. (**c**) Pipeline for the analysis. AD: Alzheimer’s Disease; HC: healthy control; MCI: mild cognitive impairment; PD: Parkinson’s disease; Neuroimaging in Frontotemporal Dementia (NIFD); nADrp represents all of the following groups (HC, PD and FD); ADrp is made up of MCI and AD groups; ROI: region of interest. LASSO: least Lasso or elastic net regularization for linear models. GLMLASSO: General Linear Model with Least Absolute Shrinkage and Selection Operator (LASSO) regularization. MIRIAD: Minimal Interval Resonance Imaging in Alzheimer’s Disease. AIBL: Australian Imaging Biomarkers and Lifestyle.ROI: region of interest. ROC: receiver operating characteristic.

In this study, all clinical and image data were downloaded from the ADNI website (adni.loni.usc.edu). For training and testing datasets, 1592 subjects with 3T MRI scans from four open access datasets were adopted for machine learning model development. In addition to participants evaluated for AD, individuals from the Parkinson’s Progression Markers Initiative (PPMI) Parkinson’s disease (PD) cohort and the Neuroimaging in Frontotemporal Dementia (NIFD) cohort were included, reflecting the spectrum of neurodegenerative disorders commonly encountered in memory clinic settings. The inclusion of additional pathologies would further enhance the clinical applicability and generalizability of the study. We contend, in our previous study ^13^ (using 1.5T) and in this one, a need to include patients in the real clinical setting who may visit the memory clinic, including PD and frontotemporal dementia, and eventually found not to have AD diagnosis. This way, true pathology is distinguished from other brain conditions. Regarding nomenclature the MCI subjects have clinical dementia rating (CDR) of 0.5, while the AD subjects were defined as CDR ≥ 1.Participant demographics, including the number of subjects in each group, age, and sex, are presented in Table 1 and depicted in Fig. 1a,b.

**Table 1:** Age and biological sex of the subjects included for nADrp versus ADrp model development.

Group	Total number of subjects (n)	Number of male (n)	Age (year, mean ± std)	Magnetic field strength
AD	283	155	74.77 ± 8.13	3.0T
HC	372	163	72.78 ± 7.73	3.0T
MCI	365	203	73.36 ± 8.12	3.0T
NIFD	275	138	63.10 ± 7.43	3.0T
PD	245	158	67.95 ± 5.78	3.0T
AIBL (timepoint 1)	35	14	72.71 ± 6.72	3.0T
AIBL (timepoint 2)	35	14	74.37 ± 6.70	3.0T
AIBL (timepoint 3)	35	14	75.89 ± 6.58	3.0T
AIBL (timepoint 4)	35	14	77.29 ± 6.62	3.0T
AIBL (timepoint 5)	35	14	78.71 ± 6.54	3.0T
ADNI 1.5T 1st	107	60	75.70 ± 7.19	1.5T
ADNI 1.5T last	107	60	79.41 ± 7.37	1.5T
MIRIAD 1st scan	69	30	69.45 ± 7.00	1.5T
MIRIAD last scan	69	30	70.85 ± 6.96	1.5T

Figure 1a shows the ADNI dataset which include 1592 subjects with 3T T1-weighted MRI. Figure 1b illustrates the age and gender of the dataset. Significant (p < 0.05) age differences between male and female were found in HC subject groups (Fig. 1b). The x-axis of Fig. 1b shows different subject groups, while the y-axis is the age (year). In Fig. 1c, the data analysis pipeline for building and evaluating classification models are given. The arrow indicates the processing order. Yellow blocks indicate image and feature processing, which need to be done once only. The green blocks denote the steps for developing the nADrp versus ADrp model and MCI versus AD model. The requirement to conduct these steps for both models indicates that they are repeated steps (one for nADrp versus AD model, and the other for MCI versus AD model).

### Three external longitudinal test datasets

Three external longitudinal test datasets were used for model validation purposes; the first was the Australian Imaging Biomarkers and Lifestyle (AIBL) longitudinal dataset ^34^, that assessed 35 subjects with 3T MRI scan repeatedly for 6 years (Table 1). The AIBL study methodology has been reported previously ^34^. In the dataset, there were 280 scans with 5-visit (5 time points) involving 70 subjects (including 35 subjects who had all 5-visit).

The control group (nADrp) for the machine learning classification model included HC, patients with frontotemporal dementia, and subjects with PD, and the disease group (ADrp) included participants with AD-related MCI or AD. The list of these subjects was included in the supplementary materials (AIBL_35_subjects_list.xlsx), which includes 5 converters and 3 reventer subjects.

The second external validation dataset included 107 subjects (60 male, HC = 42, MCI = 49, AD = 16). These T1-weighted MRIs were collected with 1.5T scanners. This was a longitudinal dataset that had two longitudinal scans; the gap between two scans were more than 2 years. The age was 75.70 ± 7.19 for the first scan, and was 79.41 ± 7.37 for the last scan (see Table 1 for details). To distinguish this dataset from ADNI training and testing datasets (1592 subjects on 3T scanner), the name of ADNI 1.5T will be used to reference this dataset.

The third external validation dataset was the Minimal Interval Resonance Imaging in Alzheimer’s Disease (MIRIAD) longitudinal data. Sixty-nine subjects (31 male; HC = 23, MCI = 7, and AD = 39) were included, and the data were collected on a 1.5T GE scanner and with multiple scans within 2 years (Table 1). There was no converter or reverter in this dataset. The first and last scans were employed to validate the model, and the maximum gap between the first and the last scans was 2.07 years. The age between nADrp (69.66 ± 7.02 for the 1^st^ time point, 71.11 ± 7.02 for the last/2^nd^ time point) and ADrp (69.34 ± 6.98 for 1^st^ time point, 70.72 ± 6.93 for the last/2nd time point) was not significant at both time points. The detailed description of the dataset can be found in reference^35^.

### MRI brain image segmentation

For model development (ADNI 1592 subjects), a high-resolution structural T1-weighted MRI from each subject was collected from 3T MRI scanners. Detailed information for each subject and scans can be traced back from the supplementary files (nADrp_vs_ADrp_1274_train_list.csv, nADrp_vs_ADrp_318_test_list.csv). For the AIBL datasets, 70 subjects (AIBL_70_subjects_longitudinal_scan_1st_scan_List.csv) with several longitudinal scans (including 35 subjects with 5 scans) were also included in the supplementary materials (AIBL_35_subjects_list.xlsx). For the ADNI 1.5T dataset, a list of subjects (ADNI_1dot5_T_subject_scan_list.csv) was also included. From the subject and image identification number, the scan information can be found from ADNI website.

For the MIRIAD external testing dataset, all scans were conducted on the same 1.5T Signa MRI scanner (GE Medical systems, Milwaukee, WI) and acquired by the same radiographer. Three-dimensional T1-weighted images were acquired with an IR-FSPGR (inversion recovery prepared fast spoiled gradient recalled) sequence. The imaging parameters were as follows: field of view, 24 cm; matrix size, 256 × 256; 124 coronal partitions with a slice thickness of 1.5 mm; repetition time (TR), 15 ms; echo time (TE), 5.4 ms; flip angle, 15°; and inversion time (TI), 650 ms. A complete list of subjects and scans is provided in the supplementary materials.(MIRIAD_subject_scan_list.csv).

Three desktop computers with Intel ® CPU with Ubuntu 18.04 system were used for the image processing and analysis from May 2022. The first computer has Xeon ® CPU E3-1241 v3 with frequency of 3.50 GHz. The CPU has eight cores and the RAM is 32 GB. The second has Core (TM) i7-6850 K CPU with frequency of 3.60 GHz and 12 cores; the computer has 56 GB RAM. The third has a Core (TM) i7-7820X CPU with frequency of 3.60 GHz; it also has 16 cores with 64 GB RAM. Python (https://www.python.org/, version 3.8 and version 3.10) with multi-processing method was used to run FreeSurfer in parallel on these three desktop computers.

The FreeSurfer (https://surfer.nmr.mgh.harvard.edu/^27^, version 7.3.2, freesurfer-linux-ubuntu18_x86_64-7.3.2-20220804-6354275) was used, and the FreeSurfer *recon-all* command was applied to obtain the segmented brain image and associated brain mask regions for radiomics feature extraction. The FreeSurfer reconstruction results were compared based on one randomly selected subject, and the analysis was carried out after confirming that there was no difference between different FreeSurfer reconstruction results from different computers. Radiomic features were extracted using TexLab (v2) within MATLAB® (version 2022b) (https://uk.mathworks.com/). Feature extraction was conducted across all grey-level discretization settings, including 4, 8, 16, 32, 64, 128, and 256 grey levels. Python programs were developed to run MATLAB via the Python engine.

Learning curves for the LASSO classification models were generated on a high-performance computing (HPC) system to evaluate model stability and potential overfitting. Models were trained using varying fractions of the training dataset ([0.1, 0.2, 0.4, 0.6, 0.8, 1.0]). Each experiment was repeated 10 times per fraction, and the mean performance with standard deviation was calculated to derive error bars.

### Radiomic feature extraction

The detailed pipeline for the data analysis is shown in Fig. 1c. As first step in the image analysis, FreeSurfer was employed to parcellate the brain into different regions. All surface of ADNI 1592 subjects, 280 scans (from AIBL, including all longitudinal scans), 214 ADNI 1.5T images (107 subjects with 2 longitudinal scans), and 138 MIRIAD image (69 subjects with 2 longitudinal scans) data were reconstructed with FreeSurfer *recon-all* function. From the results of FreeSurfer, a skull-stripped brain image (brain.mgz) and associated brain region segmentation masks (aseg.mgz) were used for feature extraction ^13^. At first, 115 brain regions were included in the analysis ^13^, but because of the instability of some brain regions returning NaN (not a number) value, only 107 brain regions were used for feature extraction. The names of these regions can be found in the supplementary materials (FreeSurfer_Regions_List.csv). For each selected brain region, 3906 radiomic features were extracted with TexLab v2 and a correlation statistic was adopted to remove any correlated radiomic features, i.e., any features with correlation coefficient larger than 0.95. The remaining features were combined from the different brain regions, and 136,112 features were employed for model feature selection.

### Feature normalization and training and testing data split

A z-score method ($z = \frac{x - \mu }{\sigma }$) was applied for feature normalization, where *μ* is the mean value of the feature, and *σ* is the standard deviation. If the feature does not change across subjects, i.e., the feature value for each subject is the same, the *σ* is 0. If normalization of this radiomic feature results in a NaN value, the feature is excluded from further analysis. .  After that data were split into training and testing data (stratified by event: ADrp for classifier1 or AD for classifier2). The dataset was divided using a stratified hold-out scheme, with 80% of subjects assigned to the training set and 20% to an independent test set. The data splitting was performed using MATLAB code as follows: c = cvpartition(yTrain,’HoldOut’,0.2,’Stratify’,true) (https://uk.mathworks.com/help/stats/cvpartition.html). Region-of-interest (ROI)–based brain features derived from 107 predefined regions were analyzed for 1,592 subjects. Diagnostic labels were binarized into a nADrp group, comprising cognitively normal, PD, and NIFD cases, and an ADrp group, comprising MCI and AD. All features were standardized using z-score normalization. 

To prevent information leakage, all feature screening procedures were conducted exclusively within the training data. Feature robustness was evaluated using a two-stage stability analysis based on internal resampling of the training set. First, repeated random subsampling was performed to generate paired training subsets, and two-sample t-tests were applied to each feature to assess the consistency of mean values across subsets. Features exhibiting significant mean differences (p ≤ 0.05) were classified as unstable and excluded. Second, for features retained after mean stability screening, two-sample F-tests were used to evaluate variance consistency across resampled subsets; features with significantly different variances (p ≤ 0.05) were similarly classified as unstable.

To quantify feature instability, the resampling procedure was repeated 1,000 times, and the frequency with which each feature was identified as unstable was recorded. This frequency was used as a quantitative measure of feature robustness across data partitions. Only features demonstrating stable mean and variance properties across resampled training subsets were retained for downstream model development and final evaluation on the held-out test set. To mitigate false positives introduced by the resampling procedure, False Discovery Rate (FDR) control using the Benjamini–Hochberg method was applied to the feature-wise statistical tests within the training set. Feature stability across repeated resampling was also evaluated, and most selected features were found to be consistently identified, suggesting that the results are not driven by random variation. Feature-level statistical tests were applied as an initial screening step to reduce dimensionality within the predictive modelling pipeline. These tests were used as heuristic tools to prioritize candidate features and were not intended to support formal statistical inference or establish biological associations. All downstream conclusions were based on model-level performance rather than feature-level significance.

The mean and standard deviation of the selected features were implemented for normalizing features from (external) testing datasets. For independent external testing datasets, features from TexLab v2 were extracted in the same way as training data. The feature names (and brain regions) from training dataset were used to localize these features from the external testing datasets.

Models for nADrp versus ADrp (classifier 1) and MCI versus AD (classifier 2) classification were developed by the same approach. For nADrp versus ADrp classification model, 1592 cases with 3.0T T1-weighted MRI image data from ADNI database were split into training (n = 1274, 80% of 1592) and testing (n = 318, 20% of 1592) data, with random stratified splitting (to dependent variable, i.e. nADrp or ADrp subject) and models were built based on the training dataset. For MCI versus AD classification model, 543 (80% of 678) subjects were included as training subjects, and 135 (20% of 678) subjects were used as testing subjects.

### Machine learning models for classification

Machine learning classification models were built with MATLAB statistics and machine learning toolbox (Version 12.4). MATLAB function LASSO/glmlasso.m (https://uk.mathworks.com/help/stats/lasso.html) was applied to select features from training data^36^. The MATLAB code was : [B,FitInfo]=lassoglm(XTrain,YTrain,’binomial’,’CV’,10). For the LASSO method, optimal features/model was determined by minimizing the L1 norm. A ten-fold cross validation method was applied for the classification. A binomial distribution was selected to implement logistic regression. We selected the features corresponding to the regularization parameter reached at the minimum deviance (Figure S1). The features and their corresponding weights/coefficients were used to determine the weighted sum value.

The default threshold for predicting nADrp versus ADrp is 0.5 (probability from logistic regression), with the prediction probability ranging between 0 and 1. From logistic regression, we have:

$$p = \frac{1}{{\left( {1 + e^{{ - \left( {weightedSum + b_{0} } \right)}} } \right)}}$$

where *p* is probability $,b_{0}$ is intercept, and the weighted sum has the following relation with the probability:

$$weightedSum = - ln\left( {\frac{1}{p} - 1} \right) - b_{0}$$

All possible thresholds were considered, using a simple MATLAB code, to achieve the optimum accuracy based on the training dataset. The threshold should approach 0.5 given a sufficiently large and balanced dataset. We then applied this exact threshold to all test datasets. The same trained model (cross-sectional data) was used for all longitudinal datasets.

### Machine learning model performance criteria

After models were established, training and (external) testing datasets were used to evaluate the performance of the models. Based on the optimal probability threshold, the area under the curve (AUC) of the receiver operating characteristics (ROC) , F1, the confusion matrix, positive predictive value (PPV), negative predictive value (NPV), and the weighted sum classification value were applied to testing datasets for assessing model performance. The calculation of PPV, NPV, and weighted sum can be found in the supplementary materials.

In the method, the random splitting training and testing data could affect the final model selection. In addition, because the cross-validation method was adopted, the final models could have a degree of subjectivity. The models were selected after repeating the calculation several times.

## Results

### FreeSurfer segmentation results

**Fig. 2:**
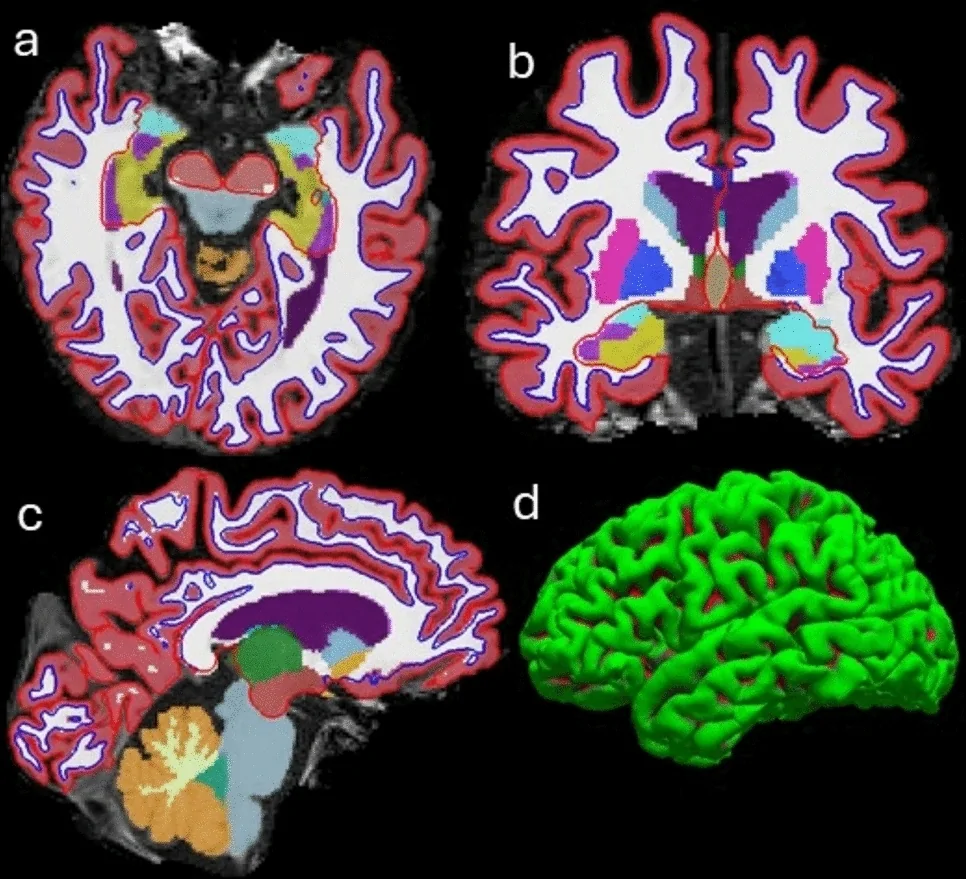
A typical FreeSurfer segmentation and reconstruction results from an 82 year old female subject with MCI: (**a**) transverse plane, (**b**) coronal plane, (**c**) sagittal plane. (**d**) 3D surface of this subject.

Figure 2a–d show one example of brain masks obtained from FreeSurfer for radiomic features extracted from an 82 years’ old female MCI subject. These images were the output of FreeSurfer with colour coding of different segmented brain regions. Figure 2a–c show the same subject viewed from transverse, coronal, and sagittal plane, respectively, while Fig. 2d displays the surface reconstruction result.

### Model classification accuracy using training and testing datasets for nADrp vs ADrp (classifier 1)

Using 1274 (80% of 1592) subjects for training and adopting the regularization parameter (lambda) value that corresponds to minimum deviance value for the regularization (Figure S1 in the supplementary materials), a vector with 363 features was determined by the LASSO method for classifying nADrp versus ADrp. The nADrp group includes HC, PD, and NIFD subjects (Fig. 1a), while ADrp includes MCI and AD patients. After applying a tenfold cross validation LASSO method for selecting the optimal model, the regularization parameter lambda, corresponding to the minimum deviance value, and the optimal number of features included in the model were determined (Figure S1).

**Fig. 3:**
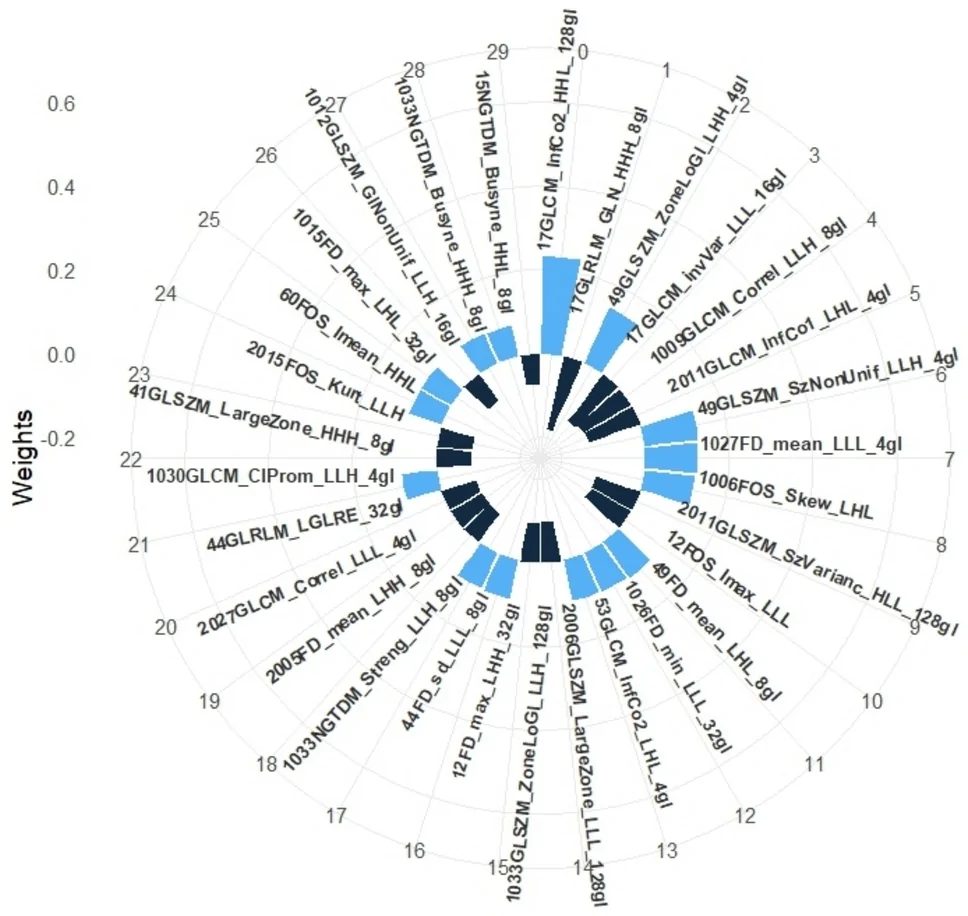
A Phylogenic tree plot of 30 radiomics features with largest weights magnitude in the nADrp versus ADrp model.

Figure 3 shows a circular plot for thirty features with largest magnitude weight in the nADrp versus ADrp classification model. The blue colour denotes the positive weight in the model, black shows the negative weight in the model. Feature number 0 has the largest weight magnitude, while feature number 29 has the smallest magnitude weight in the figure. The number in front of each radiomic feature name denotes the brain region number, followed by the radiomics name. From this number, the name of the brain region can be found from (FreeSurfer_Regions_List.csv).

The LASSO method selected 363 features (model_nADrp_vs_ADrp_363_features.csv in the supplementary materials) for nADrp and ADrp classification model. The four largest weight magnitude was derived from the left hippocampus, and the right thalamus, respectively. The highest-ranking feature was 17GLCM_InfCo2_HHL_128gl. As a wavelet-filtered Gray-Level Co-occurrence Matrix (GLCM) Information Measure of Correlation 2, it captures the complexity of spatial intensity relationships and reflects hippocampal microstructural heterogeneity, potentially linked to variations in cellularity, necrosis, stromal architecture, and vascular organisation.

**Fig. 4:**
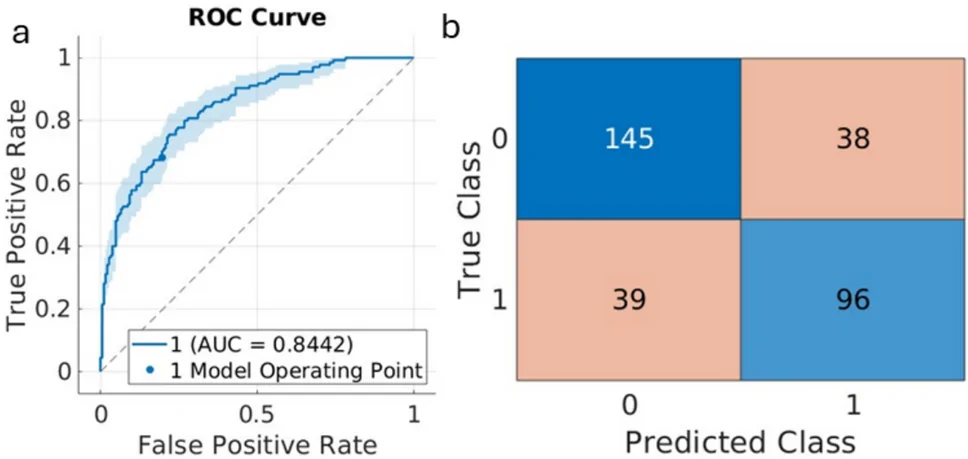
Classification metrics for the nADrp and ADrp classification model from testing datasets. (**a**) AUC curve from testing dataset. (**b**) Confusion matrix from testing dataset.

Based on the selected model, the optimal cut-off probability for nADrp and ADrp classification can be obtained using the training data with best classification accuracy. The optimal threshold obtained from training dataset was 0.4579, which was applied to all testing and validation prediction. Using the cut-off probability, the AUC of ROC (Fig. 4a) obtained from testing dataset, and associated confusion matrix (Fig. 4b) for the classification can be calculated and displayed. Figures S2 a/b show the results of AUC and confusion matrix from training dataset. The number of subjects and AUC value are included on the figures, and the other additional classification evaluation criteria are included in Table 2. The weighted sum plots (Figure S2) for training (Figure S2 c) and testing data (Figure S2 d) can be found in supplementary materials.

**Table 2:** Classification criteria for nADrp versus ADrp model.

nADrp vs ADrp	Sensitivity	Specificity	Accuracy	F1	NPV	PPV
Training	0.9705	0.9726	0.9717	0.9670	0.9780	0.9634
Testing	0.7111	0.7923	0.7579	0.7138	0.7880	0.7164
AIBLI 1st	0.5000	0.8966	0.8286	0.5000	0.8966	0.5000
AIBL 2nd	0.6667	0.8276	0.8000	0.5333	0.9231	0.4444
AIBL 3rd	0.7143	0.7857	0.7714	0.5556	0.9167	0.4545
AIBL 4th	0.6250	0.7037	0.6857	0.4762	0.8636	0.3846
AIBL 5th	0.8750	0.6667	0.7143	0.5833	0.9474	0.4375
ADNI 1.5T 1st	0.8154	0.6429	0.7477	0.7970	0.6923	0.7794
ADNI 1.5T last	0.9231	0.4524	0.7383	0.8108	0.7917	0.7229
MIRIAD 1st	0.9130	0.7391	0.8551	0.8936	0.8095	0.8750
MIRIAD last	0.9565	0.7826	0.8986	0.9263	0.9000	0.8980

Classification model for nADrp versus ADrp using 363 features with probability threshold of 0.4579.

**Fig. 5:**
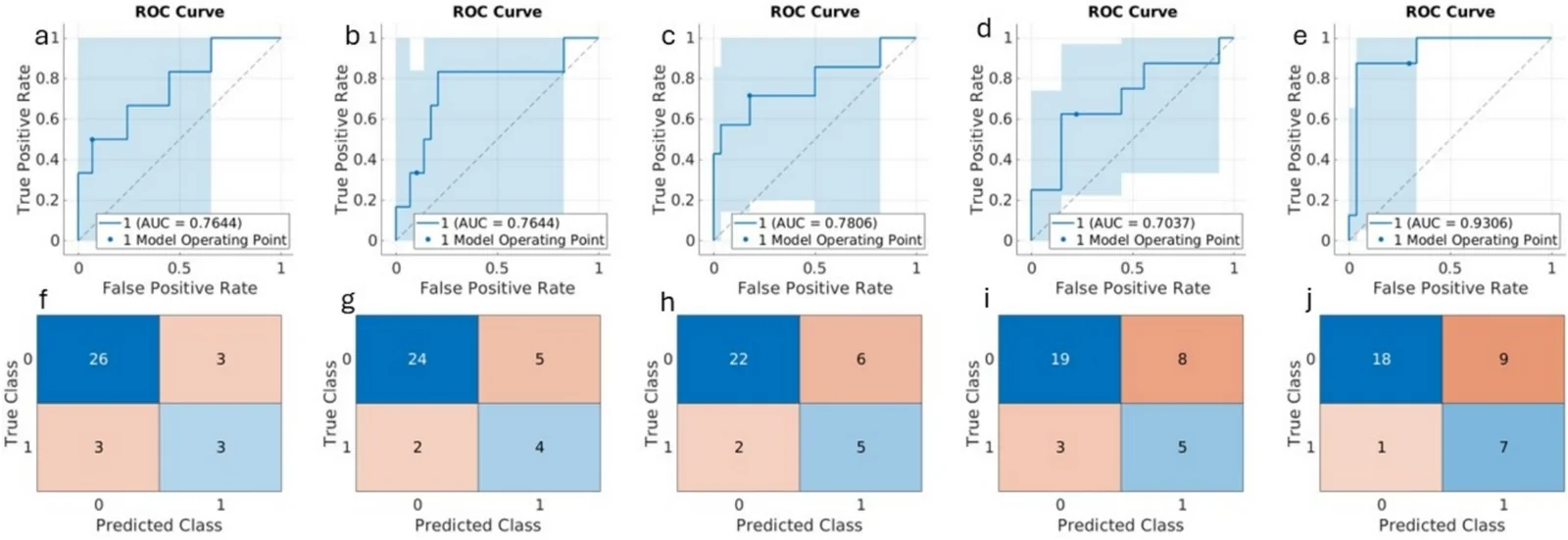
External validation results using AIBL longitudinal datasets. (**a**–**e**): AUC of ROC plots. (**f**–**j**) Confusion matrices.

Using the selected model and associated probability cut-off value, the AIBL longitudinal dataset was adopted to evaluate the model with 363 features externally. Figure 5 shows the results from the 1st scan to the 5th scan. Figure 5a–e are the AUC/ROC curves, and Fig. 5f–j are the confusion matrixes for the classification. Figure a/f, b/g,c/h,d/i, and e/j shows the results from the first visit to the 5^th^ visit longitudinal scans, respectively. The number of subjects in each validation can be calculated from confusion matrix or weighted sum plot (Figure S3).

For nADrp versus ADrp classification model, Table 2 (nADrp vs ADrp) shows the specificity accuracy, F1, NPV, and PPV values for Fig. 4, Figure S2 a/b, and Fig. 5. Based on the optimal probability cut-off value (0.4579), the classification performance criteria were calculated. For AIBL external testing dataset, with increase of disease progression except the 4th visit (Fig. 5d), there was an increase of sensitivity, accuracy, and F1 for the longitudinal external testing datasets.

The genetic information for these 35 subjects was obtained, and the prediction results based on nADrp versus ADrp model was included in the supplementary materials (AIBL_35_subjects_list.xlsx). From this longitudinal and genetically profiled cohort, the model predicted 6-year outcome in patients with APOE epsilon status in all but one subject (subject ID 73).

### ADNI 1.5T and MIRIAD external testing results using nADrp versus ADrp model

**Fig. 6:**
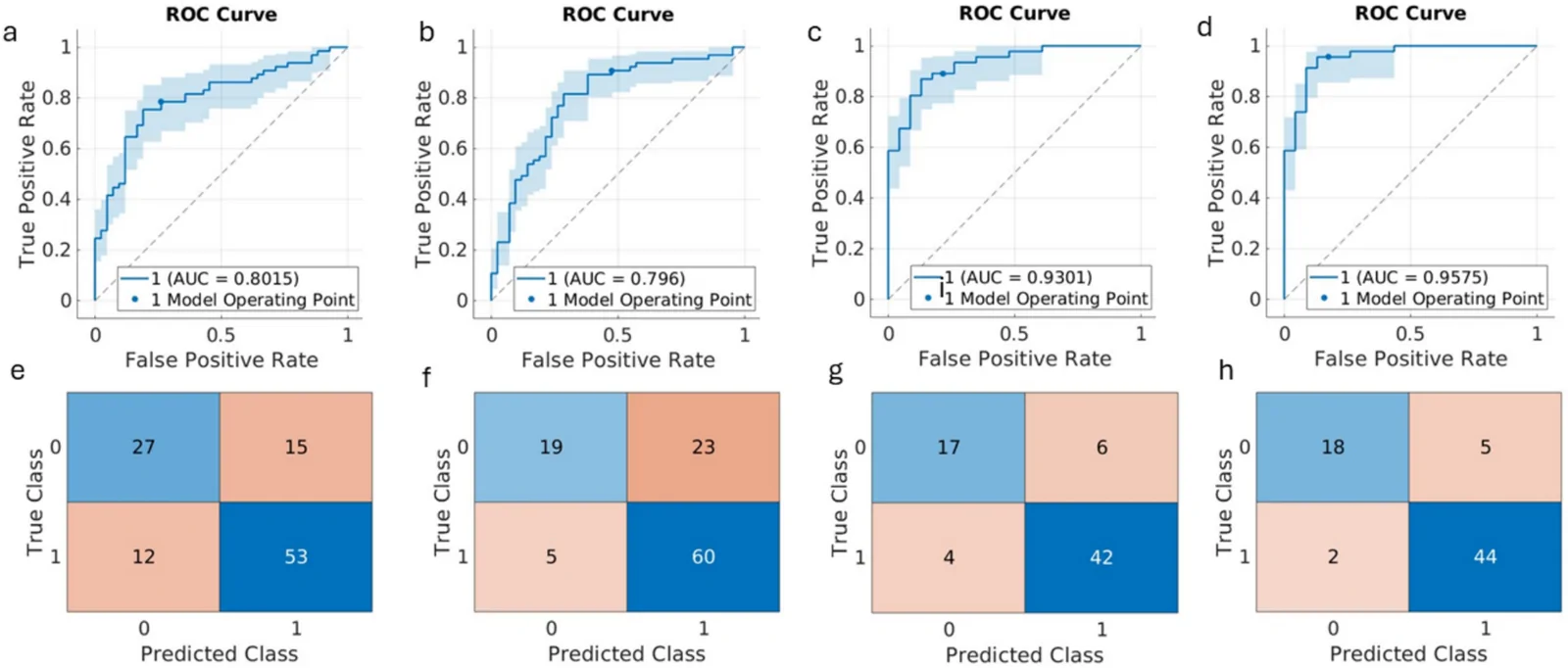
nADrp versus ADrp classification model with 363-features testing results on ADNI 1.5T (**a**,**b**,**e**,**f**) and MIRIAD (**c**,**d**,**g**,**h**) datasets. (**a**–**d**) AUC of ROC plots. (**e**–**h**) Confusion matrices.

Based on the nADrp versus ADrp model, ADNI 1.5T and MIRIAD data were employed to evaluate the model performance, and the results are shown in Fig. 6. Figure 6a,b,e,f were obtained from ADNI 1.5T testing data, while Fig. 6c,d,g,h were calculated from MIRIAD dataset. Figure 6a/e and c/g were generated from the 1st scan, Fig. 6b/f and d/h were computed from the last longitudinal scan from ADNI 1.5T and MIRIAD dataset, respectively. The associated weighted sum plot can be found in supplementary materials (Figure S4).

From the ADNI 1.5T dataset result, there was no obvious AUC value change between two scans (comparing Fig. 6a and b, Table 2), while for MIRIAD dataset, the AUC value increased from 0.93 to 0.96 between two longitudinal scans (comparing Fig. 6c and d, Table 2).

### MCI versus AD classification model

The total number of subjects, number of male subjects, and age of these subjects included for MCI versus AD classification model can be found in Table 3.

**Table 3:** Age and biological sex of the subjects included for MCI versus AD model development.

Group	Total number of subjects (n)	Number of male (n)	Age (year, mean ± std)
ADNI AD	283	155	74.77 ± 8.13
ADNI MCI	365	203	73.36 ± 8.12
AIBL (visit 1)	18	12	72.72 ± 6.65
AIBL (visit 2)	15	8	74.73 ± 5.95
AIBL (visit 3)	14	7	77.43 ± 6.08
AIBL (visit 4)	15	7	78.47 ± 5.46
AIBL (visit 5)	8	3	79.88 ± 6.60
ADNI 1.5T 1st	65	38	75.22 ± 7.56
ADNI 1.5T last	65	38	78.63 ± 7.56
MIRIAD 1st scan	46	19	69.34 ± 6.98
MIRIAD last scan	46	19	70.73 ± 6.93

**Fig. 7:**
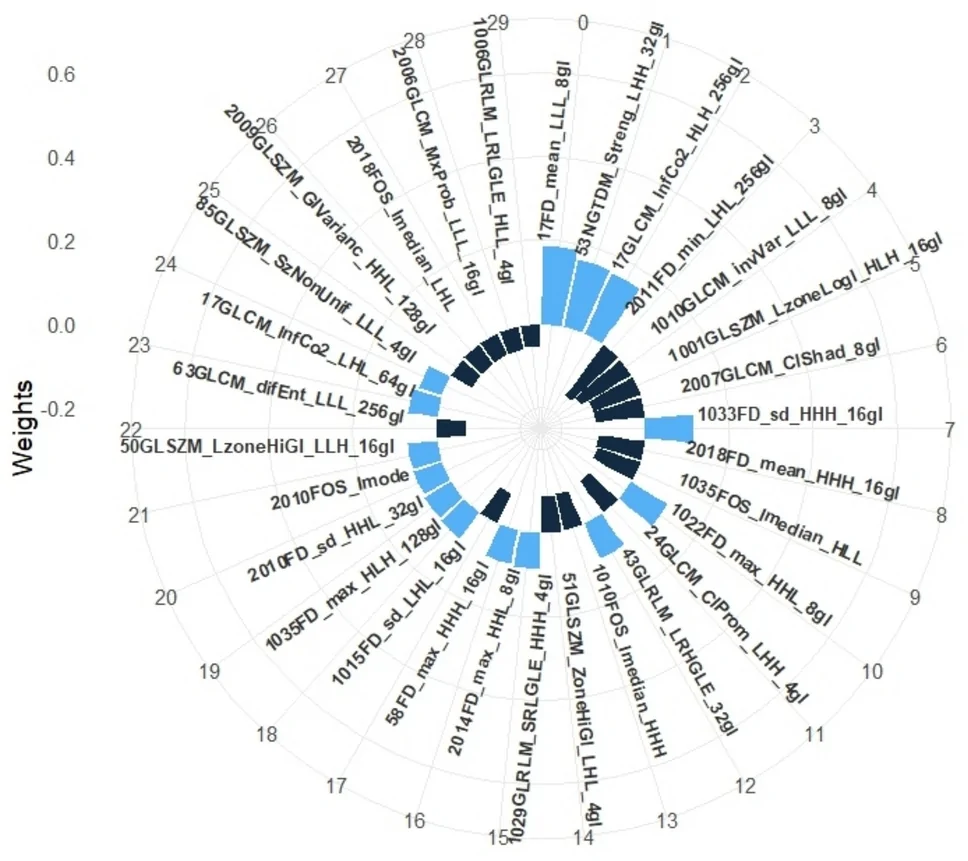
Discrimination of MCI versus AD. Circular plots for the thirty features with largest magnitude weight in the MCI versus AD classification model.

Based on 678 training dataset and the MATLAB model selection (glmlasso.m) function, the LASSO model selection lambda value was determined (Figure S5). After applying LASSO, 132 radiomics features were selected for MCI versus AD classification model. Thirty radiomics features with the largest magnitude weight from the final selected model are displayed in Fig. 7. The largest three weights (magnitude) were derived from the left and right hippocampus. In Fig. 7, the blue colour denotes the positive weight in the model, black shows the negative weight in the model. Feature number 0 has the largest weight magnitude, while feature number 29 has the smallest magnitude weight. The number in front of each radiomics feature name denote the brain region number, followed by the radiomics name. From this number, the name of the brain region can be found from (FreeSurfer_Regions_List.csv), and the details of these radiomic features can be obtained from the supplementary materials (model_MCI_vs_AD_132_features.csv).

**Fig. 8:**
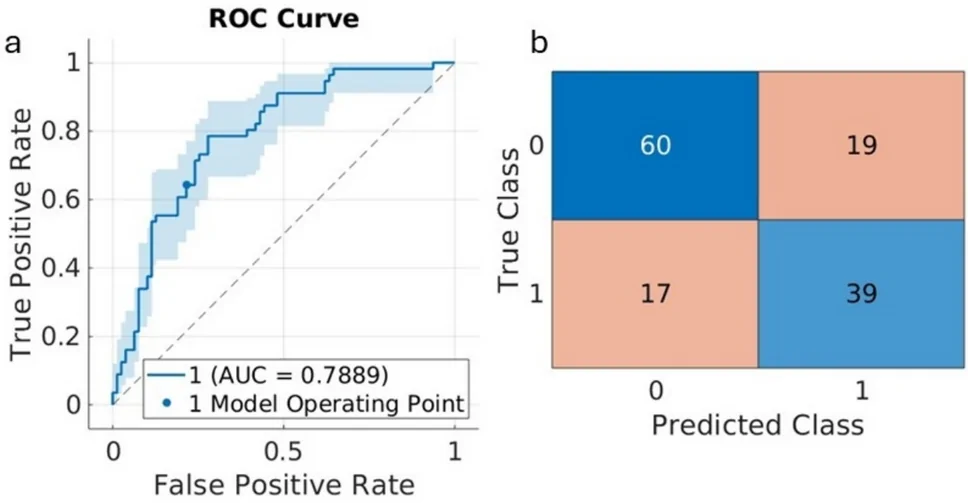
Classification metrics for the MCI versus AD model from testing datasets. (**a**) AUC curve from testing dataset. (**b**) Confusion matrix from testing dataset.

Based on the MCI vs AD model with 132 features, Fig. 8 shows the classification performance results based on testing datasets. The AUC, confusion matrix, and the weighted sum from training and testing data can be found from supplementary materials (Figure S6). Similar to nADrp versus ADrp model, the optimal probability cut-off value was calculated to be 0.4229. This cut-off value was applied to the testing and external validation studies. The other model performance criteria such as F1 value can be found in Table S3. The validation results based on AIBL and MIRIAD external testing datasets can be found in the supplementary materials (Figures S12 and S13).

### Classification accuracy for reverter and converter subjects

To study how disease progression influences classification accuracy, we selected two subgroups from AIBL longitudinal dataset for the evaluation. The first group was the reverter patients defined as those who revert from a CDR scale score of 0.5 to CDR 0 ^37^. A previous study showed that reversion is common when tracking change from the classification of MCI back to normal cognition ^38,39^. Our study recorded three subjects who have CDR of 0.5 from the beginning of study, while at the end of study, the CDR become 0 (become nADrp subject). The classification results using the nADrp and ADrp model was presented in supplementary information (Table S1) and showed disconnect between radiomics predicted outcome and CDR scores.

The second converter group is defined as asymptomatic adults who convert to MCI ^40^. From AIBL database, there were 5 subjects who have CDR of 0 (normal subjects) at the beginning of study, but at the end of the study, the CDR was 0.5. The weighted sum box plot for 5 converter (from normal to MCI) subjects from time point 1 to 5 was presented in the supplementary information (Table S2). For the nADrp versus ADrp classification model, the results showed prediction outcome increases with disease progression.

## Discussion

We have developed two machine learning classifiers for predicting ADrp from nADrp (classifier 1) or AD from MCI (classifier 2) using solely 3T T1 MRI radiomics. Both models embody automatic segmentation and extraction of radiomics features from T1-weighted MRI. The effectiveness of these models was evaluated on internal testing data and three longitudinal external datasets, including 2 datasets collected on a 1.5T scanner. Comparing the classification accuracy of our initial models based on training and testing datasets for nADrp and ADrp classification, there was a reduction of AUC, suggesting that the distribution of two datasets was different. To reduce the effect of radiomic features differences, mean and variance of radiomic features from training data were split into two groups and compared. The features with significant differences of mean and variance were defined as less stable features and were removed before applying the LASSO method for model selection. Classification models with stable features were employed, and it turned out that the selected model with stable features was more concise and had less features included in the model, compared to the model without removing the less stable radiomics features. Furthermore, sensitivity analyses employing the LASSO across multiple training splits demonstrated that, while the specific feature composition varied, hippocampal and limbic features were consistently retained, supporting the robustness of biologically meaningful signals.

### nADrp versus ADrp model

From the longitudinal AIBL external testing data study, the classification accuracy value in terms of AUC value showed a wide range, i.e., from 0.76 for the 1st scan to 0.93 for the last scan of the external longitudinal testing dataset (Fig. 5), perhaps expected from a model trained to discriminate wide ranging disease phenotype (ADrp: MCI to AD) from not AD phenotype (nADrp). The result suggested that the model has higher classification performance for the patients at later stage of disease development. This may also explain the classification accuracy difference from previous studies, as these methods may be tested on the MRI collected at different stage of AD progression. Compared with other previous studies ^41,42^, the simple and objective nADrp versus ADrp classification model achieved a similar classification accuracy. For example, based on 2-D image method, a recent study achieved 99% HC and AD classification accuracy ^42^. This method needs to select the 2-D image from 3D image volume. However, this approach does not consider the real clinical setting where 3D MRI is often acquired for patients’ diagnosis. In addition, this method adopted an adaptive synthetic sampling approach to resolve imbalanced input-dataset, which again deviates from real clinical settings. Our method is full automatic, and it is similar to previous studies ^13,22,43^ in which radiomics features were obtained from FreeSurfer. With respect to class imbalance, stratified splitting was used to preserve class proportions where feasible; however, we acknowledge that stratification alone cannot fully mitigate extreme imbalance in small external cohorts. Techniques such as Synthetic Minority Over-sampling Technique (SMOTE) or class weighting were considered, but were not applied in the external validation analyses in order to avoid introducing synthetic samples or altering the original data distribution, which could further complicate interpretation in these small cohorts.

Using external longitudinal datasets, AIBL, we discovered that the stage-shift influences model performance. We found that the machine learning methods perform better at later stages of disease progression. From the last time point of the AIBL testing dataset, AUC of the classification model was high, suggesting that the trained dataset was similar to the later stage of AD progression. This result was in agreement with a previous study on a longitudinal dataset ^44^. This was also true for the performance evaluation from MIRIAD dataset (Fig. 6c,d), where there were slightly increased AUC values between two longitudinal scans.

However, for ADNI 1.5T data validation study (Fig. 6a,b), the nADrp versus ADrp model produced similar results between two longitudinal scans in terms of AUC values; this could be due to the stability of the disease within the study period, suggesting that the classification accuracy for longitudinal datasets is contextual and depends on the study cohort used.

### Prediction performance differences from 3 datasets

We have focused on 3T MRI training and applied this model to both 3T and 1.5T data. We have considered two classifiers, 1 and 2, to simplify analysis and discussion. Performance increased in two datasets with AIBL (72 months) and MIRIAD (24 months). For ADNI 1.5 dataset, the performance was stable (48 months). This is likely due to the slow progression of the disease, making changes in pathology not detectable within this period; this is like the slow decline in cognition with disease progression. Regarding early detection, we are limited by data available to us as the CDR = 0.5 (MCI) represents the first category of disease progression. We detected ADrp vs nADrp with AUC = 0.84 on testing data (cross sectional data). When we consider the very early visits in our longitudinal datasets, we find that AUC is 0.76 (AIBL 3.0T), 0.80 (ADNI 1.5T), and 0.93 (MIRIAD 1.5T). Based on these findings, we surmise that our model detects early disease with reasonable to high performance.

The AUC decreased from 0.996 (Figure S2) in the cross-sectional training set to 0.84 (Fig. 4) in the test set. There may be multiple reasons for this, key amongst which is that, even though we analyzed 1274 patient’s data for training and 318 in the testing, there could have been overfitting when the penalized regression model (LASSO) was implemented. While we implemented testing on unseen datasets, we may not have fully controlled feature distribution between with performance ranging between 0.80 to 0.96 (Fig. 6).

### Converter and reverter prediction

For the converter prediction, the classifier performance was better at the 5^th^ scan time point (Table S2) than at the 1^st^ scan time point based on AIBL dataset. This was understandable as the classification performance was better at the 5th scan (Fig. 5) using AIBL dataset. However, for the reverter subjects from AIBL, the performance of the classifier was not good (Table S1), because the prediction accuracy was 1/3 at the 1st and 5th time points of the MRI scan. To improve the prediction accuracy for reverter subjects’ classification, that is if they are confirmed in longitudinal studies, a new model based on the reverter datasets exclusively needs to be developed to address this problem.

### MCI versus AD model

Based on the same approach for developing nADrp and ADrp classification model, we have proposed a classification model for MCI and AD subjects, which in practice will permit two-step classification of nADrp or ADrp and further distinction of the latter into MCI or AD. The model has 132 stable radiomics features, and the model was evaluated on internal and longitudinal external datasets. Again, the model classification accuracy varied between the 1st scan (AUC = 0.59, Fig. S12a ) and 2nd scan (AUC = 0.93, Fig. S12b) using AIBL dataset.

For the ADNI 1.5T and MIRIAD external testing data results (Fig. S13), the AUC varied between 0.56 (Fig. S13c) and 0.71 (Fig. S13d). For the MIRIAD dataset, with AD progression, the trend of increased classification performance over time did exist. However, there was a decreased AUC between two longitudinal scans from ADNI 1.5T dataset (comparing Fig. S13a and b). These results suggest that the classification model accuracy depends on the datasets, and the trend of increased classification performance over time was inconsistent.

### Comparison with other models

For the MCI and AD classification model, our method is better than the current available methods with AUC of 79% from internal testing dataset (Fig. 8b), as most of current methods can only achieve about 73% of AUC ^45^. Combining with a deep learning method and cerebrospinal fluid (CSF) features from positron emission tomography (PET) image, the AUC of MCI and AD classification was 70% ^46^. Using PET and MRI with random forest ensemble method, the AUC of 65% for AD and MCI classification was achieved ^47^. While the other study achieved 89% of AUC with PET image from whole brain features for AD and later stage of MCI classification ^48^. The lower accuracy is largely due to binarizing a disease phenotype that is in reality a continuum.

As the results suggest, one factor influencing the model classification accuracy is the feature distribution of the new external testing dataset from a different geographical setting. Therefore, it is necessary to compare the feature distribution between the training dataset and the new external testing dataset. If the distribution is different, then the model prediction accuracy based on (external) testing dataset will be low. There are factors which may affect the model prediction accuracy. For example, the features were acquired from different scanners. A recent study showed that there were scanner differences affecting the feature values from 3T scanner ^44^. However, for the model development, the dataset and features were collected and extracted from multiple centres with different 3T scanners, thus it is expected that the model would work across different manufacturers. Also, although the models were built based on the features from 3T scanners, ADNI 1.5T and MIRIAD datasets testing results suggested that the model also worked for the data obtained from 1.5T scanners, as it achieved reasonable classification accuracy (Figs. 5, 6, S12, and S13).

### Biological relevance of radiomic features related to AD progression

To evaluate disease progression, longitudinal studies were adopted for AD staging. AD disease progression have been studied ^49,50^. At AD stage I to III, the lesions in the hippocampal formation worsen. In our classification models (both nADrp versus ADrp and MCI versus AD), we found several radiomics features were derived from well-known brain regions associated with AD progression. For example, hippocampus, thalamus, temporal lobe, and entorhinal cortex (EC) regions have been repeatedly reported to play an important role in stage AD stage progression ^51^^,^^52^. These selected regions were among the features selected by the modelling pipeline and may reflect patterns relevant to the prediction task; however, this should be interpreted as a model-derived signal rather than evidence of a statistically established biological association.

For the nADrp vs ADrp model, three of the largest four weight/magnitude features were derived from hippocampus and thalamus regions (Fig. 3). A few studies have demonstrated abnormalities of hippocampal regions in AD patients^22,29,53,54^. These abnormalities were observed with radiomics features in the classification model, and radiomics features were also found in previous classification model^13^, and a more recent study confirmed the hippocampal-amygdala deficits in disease progression ^55^. The third largest magnitude weight was obtained from thalamus (Fig. 3). Genetics study revealed that the thalamus region harbors abnormalities in AD patients ^52,56^. The fifth and sixth largest weights were derived from inferior temporal and lateral-occipital regions. These brain regions are also affected during the AD disease progression ^57,58^. In total, 363 features within the model were derived from 100 different brain regions.

For the MCI versus AD classification model, the largest three magnitude feature weights were obtained from hippocampus. The 39^th^ largest weight was obtained from the amygdala region. The amygdala is a critical site integrating neuromodulatory influences on memory consolidation in other brain areas ^59^. For the MCI and AD classification model, 132 radiomic features were generated from 64 brain regions, suggesting these brain regions could have been altered in the conversion of MCI to AD stage.

In our models, radiomic features derived from CSF were also highlighted. There were 4 radiomics features from nADrp versus AD model, and the 12^th^ largest weight feature in MCI versus AD model was also obtained from CSF. Another study revealed that it is useful to include MRI radiomics features derived from CSF for identifying AD subjects ^43^; a relationship between MRI CSF radiomics features and PET CSF biomarker has also been reported ^60,61^. Several studies reported that CSF is a biomarker for AD diagnosis ^45,62,63^. Particularly, a recent longitudinal study showed that changes in CSF biomarkers precede AD symptom development by up to 20 years ^62^, and the result suggests that during the 20 years preceding clinical diagnosis of sporadic AD, the time courses of CSF biomarkers diverged from that of matched cognitively normal subjects; supporting a biomarker model based on CSF for AD staging ^63^.

In summary, these radiomics features from brain regions may imply that AD-related brain abnormalities are not local but involve multiple brain regions (Figs. 3 and 7). The seed regions at the early stage of AD transmit to connected brain regions. This could happen with AD-pathology progression and with loss of functional connectivities ^64–66^, the abnormality of the brain regions spread into middle temporal lobe ^67^.

### Longitudinal study

There is increasing interest in applying longitudinal datasets for studying AD progression; a previous longitudinal study showed that cognitive deterioration was slow during the early and very late stages of AD and more rapid during the middle stages ^68^. This may indicate brain changes in the same pattern as cognitive deterioration, i.e., with rapid development during the middle stage of AD development. These variations will lead to the radiomics feature changes obtained from brain image, and consequently, will cause classification accuracy alterations using a machine learning model developed for discriminating against one disease state from another (nADrp from ADrp). This explains the machine learning classification accuracy difference between different time points of the longitudinal dataset.

A further consideration involved investigating two types of patients. The first group is the reverter group who have CDR of 0.5 from the beginning, but the CDR was 0 at the end of study. The CDR ^69^ is a global summary measure designed to identify the overall severity of dementia in which six different content areas are rated individually (memory, orientation, judgement and problem solving, community affairs, home and hobbies, and personal care). CDR are assigned on a 0–5 point scale, (0 = absent; 0.5 = questionable/very mild; 1 = present, but mild; 2 = moderate; 3 = severe; 4 = profound; 5 = terminal) ^69–71^. The CDR could fluctuate from normality (CDR = 0) to MCI (CDR = 0.5) and back during a transitional phase in disease progression between cognitive normality and dementia ^72,73^. Reverting from CDR 0.5 to 0 is a risk factor for future conversion to CDR > 0 and eventually for patients to become MCI subjects ^37^. These fluctuations pose a challenge to the classification model for patient classification. For reverter subgroup prediction, our model has 33% (one correct prediction in three cases) of accuracy for the 1^st^ and last time points of the longitudinal study (Table S1).

The second subgroup is the converter cohort, which includes 5 subjects, normal at the beginning of the study (CDR = 0), but turned to MCI subjects (CDR = 0.5) at time points 4 and 5. This trajectory indicates disease progression during the study period. The nADrp and ADrp classification model has 33% of prediction accuracy at the 1st time point, but it achieved 80% of classification accuracy at the 4th and 5th time point (Table S2). Comparing the classification at the beginning of the longitudinal study, our machine learning model achieved better classification accuracy at the end of the AIBL study. This explained our conclusion that the machine learning model performed better as the disease progressed for AIBL dataset.

The model could assist characterization of subjects with unstable cognitive condition, where CDR value is different over two repeated tests ^74^. In this situation, the CDR value can be different due to cognitive performance of the subjects. Also, the machine learning classification model could be used to evaluate new CDR methods, as there are new improved methods for CDR determination ^75^. To evaluate the effectiveness of the new CDR evaluation methods, machine learning model can be applied to predict CDR if a subject has CDR of 0 (normal subject), CDR = 0.5 (MCI), and CDR ≥ 1(AD). The machine learning method is objective and faster for CDR evaluations. It only requires a T1-weighted MRI image for the radiomics features extraction, and it is easy to implement in real clinical settings.

### Clinical implementation of the models

Applying the trained machine learning model to external dataset is challenging, especially radiomics features obtained from T1-weighted MRI under different magnetic field strengths, because the distribution of the external dataset may be different from the training dataset. Therefore, it is important to study the distribution of the external dataset before applying the model. Alternatively, the new external input data would be split into two subsets, one for testing whether the model works for the data. If so, then all the data can be applied to the model. Technically, these two models (classifiers 1 and 2) can be implemented sequentially, although they exist independently. The first stage is to identify ADrp subjects, then classifier 2 (for MCI versus AD subjects’ classification) can be applied. The most time-consuming part of implementing this method is the brain region segmentation from FreeSurfer as this will take several hours using ordinary desktop computers (Fig. 1c). The feature extraction needs to be carried out, and these features within the model need to be normalized for final prediction. The distribution of these features should be compared with the features from model training. Once these steps have been completed, it is ready to put into these radiomic features into the models for prediction in real clinical settings.

It is important to distinguish between predictive modelling and statistical inference. In this study, the primary objective was to develop and validate a model with strong generalizability. Model performance was therefore evaluated using independent and external validation datasets. Feature-level statistical tests were not used to draw inferential conclusions, and no claims are made regarding the independent statistical significance of individual features.

### Advantages of this study

There are several advantages in this study as 3 external longitudinal datasets were employed to evaluate the machine learning models. First, this study examined the longitudinal brain structural changes with machine learning methods from another viewpoint. To the best of our knowledge, there is no longitudinal study using MRI radiomics feature-based machine learning to evaluate converter and reverter subjects. We also explored the AD progression changes affecting the performance of machine learning models. Our models were validated from the external dataset in a way that closely mirrors reality in clinical settings. In contrast, a few machine learning studies have evaluated the model using external datasets ^22,42,76,77^, which is limiting because the performance of the classifier in real-world clinical settings cannot be assessed objectively.

Second, this study included comprehensive radiomic features from different brain regions for model selection. This is the largest radiomics study of AD classification to date as we included over 136,112 features from over 100 brain regions. The method is fully automatic and achieves high classification accuracy, especially for the MCI and AD subjects’ discrimination. Compared with other model selection methods such as stepwise method, the LASSO method for model selection is computational effective and efficient, it can be applied in “big data” settings, where there are many predictors in the model.

Finally, our models showed robustness, as these models were tested on 3 longitudinal datasets. Although these models were established on 3T T1-weighted MRI radiomic features, they still worked on the features extracted from 1.5T T1-weighted data (ADNI 1.5T and MIRIAD datasets, Fig. 6/S13) and 3T dataset (AIBL dataset). To increase the robustness of the model, the less stable radiomic features were excluded based on internal training dataset. These less stable radiomic features showed significant difference within the training dataset , which will lead to outliers for model estimation. The radiomic features difference could be even larger if different magnetic field strengths were applied to collect the MRI data. Previous radiomics reliability studies were conducted under different magnetic field strength ^23–26^, and reproducible radiomics features were investigated. Our results suggested that these radiomic features within the machine learning models were replicable.

### Limitations and future studies

This method was based on FreeSurfer (version 7.3.2); we found different versions of FreeSurfer produced slightly different brain image segmentation masks.Using a different version of FreeSurfer may result in variations in the extracted features used for the classification study. Due to software dependencies, our approach has been validated exclusively with FreeSurfer version 7.3.2. For instance, in 10 patients, FreeSurfer version 8.1.0 failed to generate the choroid plexus label (FreeSurfer label 31), suggesting that results may vary across software versions. Therefore, the model developed in the study was only effective when the same feature extraction software (and computer operation system) is adopted. We also found the model was sensitive to radiomics features extracted from different versions of the software. In order to replicate our results, the same version of Freesurfer should be used as we don’t fully appreciate the effect of version on brain region segmentation. If a different version of the software was applied for the analysis, attention needs to be paid to further use of the models. Moreover, it was time consuming to implement the model, as it took a long time (8 h approximately with one core of the computer used in this study) to reconstruct the surface of a single subject. FastSurfer, a deep learning method based on a convolutional neural network (https://github.com/Deep-MI/FastSurfer), can help overcome this limitation, as it performs brain region segmentation in under ~ 60 min on a GPU, significantly reducing processing time.

Second, we included the MRI data for later stages of AD development. The applicability of the model for prodromal MCI subject detection per se has not been independently evaluated. Further work needs to be carried out to evaluate the model for detecting prodromal MCI subjects.

Finally, we combined LASSO with logistic regression for building the model, and other model selection and machine learning methods such as SVM and random forest tree methods have not been explored. It should be noted that high-dimensional feature screening may introduce instability in feature selection, particularly under resampling. The use of repeated resampling strategies may lead to variability in selected features and model estimates. Besides, feature-level findings should not be interpreted as statistically robust associations but rather as components of a predictive framework. Future work will focus on validating feature relevance using dedicated inferential frameworks and independent biological or clinical studies.

## Conclusion

We have developed radiomics models for nADrp versus ADrp and MCI versus AD subject classification, based on 3T T1-weighted MRI data. We show that the models perform well across the 1.5T and 3T field strengths. Upon evaluation on both internal and longitudinal external testing datasets the results suggest that the models are robust for ADrp classification.

## Supplementary Information


Supplementary Information.


## Data Availability

All original datasets in this study were obtained from ADNI (https://ida.loni.usc.edu/) ,including ADNI dataset. The MRI image and associated clinical data can be tracked with ADNI ID numbers. All the other data supporting the findings of this study, together with the source data underlying the graphs and charts shown in the manuscript are available and have been deposited into the Mendeley database under the accession code (https://data.mendeley.com/preview/xdnxwwwv39).

## Code availability

The data analysis codes (Python and MATLAB) used to produce the results presented in this paper will be available when the paper is accepted for publishing.
