# Supplementary material for: A computational model to describe multi-regional brain architecture during neurodegeneration in Alzheimer’s disease
Source: Sci Rep. 2026 Jun 14;16:27168. doi: 10.1038/s41598-026-54109-8 (PMC13527134; doi:10.1038/s41598-026-54109-8)
Supplement: Supplementary file 1 [file 41598_2026_54109_MOESM1_ESM.docx]

***Classification NPV, PPV, and weighted sum calculations***

Positive predictive value (PPV) reflects the proportion of subjects with a positive test result who truly have the outcome of interest. Negative predictive value (NPV) reflects the proportion of subjects with a negative test result who truly do not have the outcome of interest. NPV is defined as:

$$NPV=\frac{TN}{TN+FN} \left( 1 \right)$$

while PPV or precision is defined as:

$$PPV=\frac{TP}{TP+FP} \left( 2 \right)$$

where TN is true negative, FN is false negative, TP is true positive, and FP is false positive.

***Weighted sum in logistic regression model***

The weighted sum was calculated based on the selected model. Once the model was fixed, recall logistic regression and the probability was determined as:

$$\begin{aligned} \log\left( \frac{p}{1-p} \right)=w_{1}\cdot x_{1}+w_{2}\cdot x_{2}+\cdots+w_{n}\cdot x_{n}+w_{0}\# \left( 3 \right) \end{aligned}$$

$$\begin{aligned} weighted sum=\log\left( \frac{p}{1-p} \right)-w_{0}=w_{1}\cdot x_{1}+w_{2}\cdot x_{2}+\cdots+w_{n}\cdot x_{n} \#\left( 4 \right) \end{aligned}$$

A weighted sum is defined according to equation (4), where $w_{1},w_{2},\cdots,w_{n}$ is the model coefficients/weights, $x_{1},x_{2},\cdots,x_{n}$ is the selected normalized radiomic feature, $w_{0}$ is the model intercept, and $p$ is probability.

***nADrp versus ADrp model (363 features)***

***Figure S1.*** Regularization parameter lambda (λ) changes for LASSO method model selection.

Figure S1 demonstrates the LASSO method for nADrp versus AD classification model selection. In total, 363 radiomics features were selected for nADrp versus ADrp classification model. In Figure S1, the green circle and dotted line (right vertical dotted line) locate the Lambda (y-axis) with minimum cross-validation error (FitInfo.LambdaMinDeviance = 0.01624). The blue circle and dotted line (left vertical dotted line) locate the point with minimum cross-validation error plus one standard deviation. (FitInfo.Lambda1SE = 0.02838).

***Figure S2***. a: AUC of ROC from training dataset. b: associated confusion matrix from training dataset. c and d: weighted sum plot for nADrp versus ADrp model. Training (c) and testing (d) weighted sum (see Figure 4 for associated AUC and confusion matrix plots).

***Figure S3***. Weighted sum at different time points (see Figure 5 for AUC and confusion matrix plots) using AIBL dataset. a~e: time point from the first visit to the 5^th^ visit.

***Figure S4***. Weighted sum (see Figure 6 for assocated AUC and confusion matrix plots) using ADNI 1.5T and MIRIAD longitudinal scans. a and b were generated from ADNI 1.5T dataset. c and d were obtained from MIRIAD dataset.

The weighted sum plot for ADNI 1.5T can be found in Figure S4 a (first can) and Figure S4 b ( last scan). The weighted sum plot for MIRIAD data are illustracted in Figure S4 c (first scan) and Figure S4 d( last scan).

***MCI versus AD model (132 features)***

***Figure S5***. Regularization parameter lambda changes for LASSO method for MCI versus AD model selection.

In Figure S5, the green circle and dotted line (right vertical dotted line) locate the regularization parameter lambda with minimum cross-validation error (LambdaMinDeviance = 0.0377). The blue circle and dotted line (left vertical dotted line) locate the point with minimum cross-validation error plus one standard deviation (Lambda1SE = 0.060).

***Figure S6***. Classification metrics for the MCI versus AD model from training (a, b) and weighted sum for MCI versus AD model. c: weighted sum from training dataset, d : weighted sum from testing dataset (see Figure 8 for associated AUC and confusion matrix plots from testing data).

***Figure S7***. Weighted sum for MCI versus AD model testing on AIBL dataset (see Figure 9 for associated AUC and confusion matrix plots). a~e was generated from time point 1~5, respectively.

***Figure S8***. Weighted sum for MCI vs AD model testing on ADNI 1.5T (a, b) and MIRIAD (c,d) datasets (see Figure 10 for the associated AUC and confusion matrix plots). a/c were calculated from 1^st^ longitudinal scan, while c/d was computed from 2^nd^ longitudinal scan.

***nADrp versus ADrp classification accuracy for reverter and converter subjects (with stable feature model, 363 features were selected in the model)***

***Table S1:*** predicted probability and weighted sum from the nADrp vs ADrp model for reverter group with 363 stable features. The probability threshold is 0.4579. The bolded number indicates correct prediction. The prediction accuracy for reverter is 4/15=27%.

| Subject ID (RID from AIBL) | Time point 1 (base line) | | Time point 2 (base line) | | Time point 3 (base line) | | Time point 4 (base line) | | Time point 5 (base line) | |
| --- | --- | --- | --- | --- | --- | --- | --- | --- | --- | --- |
|  | CDR | Predicted probability | CDR | Predicted probability | CDR | Predicted probability | CDR | Predicted probability | CDR | Predicted probability |
| 126 | 0.5 | 0.1096 | 0.5 | 0.1347 | 0.5 | 0.1427 | 0.5 | 0.0894 | 0 | **0.0704** |
| 38 | 0.5 | **0.5722** | 0.5 | **0.5787** | 0.5 | **0.8519** | 0 | 0.7645 | 0 | 0.8808 |
| 56 | 0.5 | 0.1767 | 0.5 | 0.3795 | 0.5 | 0.2096 | 0.5 | 0.3067 | 0 | 0.5754 |

***Table S2:*** converter subjects who have CDR=0 (normal) from the beginning of the study, while at the end, they all converted to MCI (CDR=0.5) subjects. The probability threshold is 0.4579. The bolded number indicates prediction correctness. The prediction accuracy is 17/25=68%.

| Subject ID (RID from AIBL) | Time point 1 (base line) | | Time point 2 (base line) | | Time point 3 (base line) | | Time point 4 (base line) | | Time point 5 (base line) | |
| --- | --- | --- | --- | --- | --- | --- | --- | --- | --- | --- |
|  | CDR | Predicted probability | CDR | Predicted probability | CDR | Predicted probability | CDR | Predicted probability | CDR | Predicted probability |
| 20 | 0 | **0.1817** | 0 | **0.3329** | 0 | 0.6475 | 0 | **0.3336** | 0.5 | **0.7622** |
| 33 | 0 | **0.4435** | 0 | **0.4538** | 0 | 0.7032 | 0 | **0.4567** | 0.5 | **0.7610** |
| 3 | 0 | 0.7224 | 0 | **0.5779** | 0 | 0.8444 | 0.5 | **0.5834** | 0.5 | **0.7723** |
| 570 | 0 | 0.2554 | 0 | 0.4773 | 0.5 | **0.9039** | 0.5 | **0.7433** | 0.5 | **0.8230** |
| 73 | 0 | **0.4901** | 0 | **0.2034** | 0 | **0.1922** | 0.5 | 0.3886 | 0.5 | 0.4443 |

***MCI versus AD classification using hippocampus and amygdala volumes as features***

In AD group, the hippocampus and amygdala volumes were reduced comparing to the MCI group, based on this, a logistic regression (LR) model were developed for the classification. The size of left and right side of hippocampus and amygdala were extracted from FreeSurfer output, and LR method was applied to classify AD group from MCI group.

***Figure S9.*** a: ROC curve. Classification based on only hippocampus volumes (left and right sides) and amygdala volumes (left and right sides). Using linear logistic regression method. Using the testing data, the classification accuracy is 0.61029. b:Confusion matrix obtained from testing dataset. 0:MCI, 1:AD.

The performance of this volume-based model is shown in Figure S9. Figure S9 a presents the AUC curves for the training and testing datasets, while Figure S9 b shows the corresponding confusion matrix. We can see that it has certain classification power.

***Leaning curves for the models***

In Figure S10, the left y-axis (blue curve) represents the AUC, while the right y-axis (orange curve) indicates the number of selected features. Figure S10a corresponds to the nADrp vs ADrp model, and Figure S10b corresponds to the MCI vs AD model. As shown, the selected models (363 and 132 features, respectively) fall within the stable region of the learning curves when using an 80:20 training–testing split.

***Figure S10.*** LASSO model selection learning curve. a: learning curve for nADrp vs ADrp model. b: Learning curve for LASSO model section for MCI and AD classification model. Blue curve shows the AUC of the model, while orange curve shows the number of features selected in the model.

***Model calibrations***

***Figure S11.*** Model calibration plots. Panels (a) and (b) were generated using the testing dataset, while panels (c) and (d) were derived from the UCL external validation dataset at time point 1. Panels (a) and (c) correspond to the nADrp vs. ADrp model, whereas panels (b) and (d) correspond to the MCI vs. AD model. (a) nADrp vs. ADrp model evaluated on the testing dataset (n = 318); Brier score = 0.1580.
(b) MCI vs. AD model evaluated on the testing dataset (n = 135); Brier score = 0.1827.
(c) nADrp vs. ADrp model evaluated on the UCL external validation dataset at the first time point (n = 69); Brier score = 0.1054.
(d) MCI vs. AD model evaluated on the UCL external validation dataset (n = 46); Brier score = 0.2272.

***MCI versus AD model with AIBL external testing datasets***

**Figure S12**. MCI and AD classification model performance using AIBL longitudinal external testing datasets. a~e: AUC curves; f~j: confusion matrices.

Once the MCI versus AD model has been established, AIBL longitudinal dataset was applied to evaluate the performance, and Figure S12 shows the results. These datasets were subset of 70 longitudinal AIBL dataset at different visit/scan. As most of AIBL subjects were early stage of dementia, therefore, the number of MCI and AD were small and varying (Table 3).

Figure S12 a/f, b/g, c/h, d/i, and e/j were from AIBL visis from 1 to 5, respectively. The AUC values and number of subjects can be found in Figure S12. In addition, Figure S7 in the supplementary materials shows the weighted sum of the MCI versus AD classification model. The sensitivity, specificity, accuracy, F1, NPV, and PPV values of the model classification results are included in Table 4.

**Table S3:** Classification criteria for MCI versus AD model after less stable features with significant mean and variance removed (132 radiomic features model, with probability cut-off threshold of 0.4429).

| MCI vs AD | Sensitivity | Specificity | Accuracy | F1 | NPV | PPV |
| --- | --- | --- | --- | --- | --- | --- |
| Training | 0.9251 | 0.9241 | 0.9245 | 0.9111 | 0.9450 | 0.8974 |
| Testing | 0.6964 | 0.7595 | 0.7333 | 0.6842 | 0.7792 | 0.6724 |
| AIBLI 1^st^ | 0 | 0.7059 | 0.6667 | NaN | 0.9231 | 0 |
| AIBL 2^nd^ | 1 | 0.6429 | 0.6667 | 0.2857 | 1 | 0.1667 |
| AIBL 3^rd^ | 0.8000 | 0.5556 | 0.6429 | 0.6154 | 0.8333 | 0.5000 |
| AIBL 4^th^ | 0.5000 | 0. 4545 | 0.4667 | 0.3333 | 0.7143 | 0.2500 |
| AIBL 5^th^ | 0.5000 | 0.3333 | 0.3750 | 0.2857 | 0.6667 | 0.2000 |
| ADNI 1.5T 1^st^ | 0.8125 | 0.4490 | 0.5385 | 0.4643 | 0.8800 | 0.3250 |
| ADNI 1.5T last | 1 | 0.1633 | 0.3692 | 0.4384 | 1 | 0.2807 |
| MIRIAD 1^st^ | 0.7179 | 0.2857 | 0.6522 | 0.7778 | 0.1538 | 0.8485 |
| MIRIAD last | 0.8717 | 0.2857 | 0.7826 | 0.8718 | 0.2857 | 0.8718 |

Table S3 includes sensitivity, specificity, accuracy, F1, NPV, and PPC values for the MCI and AD classification model. These results were obtained from training, testing, and longitudinal external testing datasets. The model prediction accuracy was reduced based on testing dataset. Of note, for the model prediction, the number of AD subjects is small (n<6) in the AIBL external testing datasets (Figure S12). Furthermore, the data are unbalanced, as it has more subjects for MCI group, and less subjects for AD group.

***ADNI 1.5T and MIRIAD datasets testing results***

**Figure S13**. MCI versus AD model with ADNI 1.5T and MIRIAD external testing results. a~d: AUC curves. e~h: confusion matrices.

For ADNI 1.5T dataset, 65 subjects (including 49 MCI subjects) were included (Table 3), and the results are displayed in Figure S13 a, b, e, and f. There were decreases of AUC values for the first and the last scans in the analysis (comparing Figure S13 a and Figure S13 b, Table S3). For the external testing result using MIRIAD dataset, 7 MCI and 39 AD subjects were included (Figure S13 c, d, g, h). There were increased in the performance for the first and the last scans in the analysis (Table S3). In addition, the model assessment results with weighted sum can also be found in the supplementary materials (Figure S8).
